# Effectiveness of SPECT/CT Imaging for Sentinel Node Biopsy Staging of Primary Cutaneous Melanoma and Patient Outcomes

**DOI:** 10.1245/s10434-021-10911-4

**Published:** 2021-10-26

**Authors:** Marc Moncrieff, Sarah Pywell, Andrew Snelling, Matthew Gray, David Newman, Clare Beadsmoore, Davina Pawaroo, Martin Heaton

**Affiliations:** 1 Department of Plastic & Reconstructive Surgery, Norfolk & Norwich University Hospital NHS Trust, Norwich, NR4 7UY UK; 2 Department of Nuclear Medicine, Norfolk and Norwich University Hospitals NHS Foundation, Norwich, UK; 3 Norwich Medical School, University of East Anglia, Norwich, NR4 7TJ UK

## Abstract

**Purpose:**

Coregistered SPECT/CT can improve accuracy of sentinel node biopsy (SNB) for staging melanoma. This benefit has implications for pathology services and surgical practice with increased diagnostic and surgical workload. The purpose of this study was to investigate the effectiveness of SPECT/CT imaging.

**Methods:**

SNB data were collected over a 10-year period. Preoperative SLN mapping was performed by using planar lymphoscintigraphy (LSG) for all patients (*n* = 1522) and after October 2015, patients underwent a second co-registered SPECT/CT scan (*n* = 559). The patients were stratified according to the imaging protocol. The number of nodes and nodal basins were assessed. The reasons for cancellation also were assessed.

**Results:**

A total of 95% (1446/1522) of patients underwent a successful SNB procedure. Significantly more sentinel nodes were identified by the SPECT/CT protocol (3 vs. 2; *p* < 0.0001). More patients were cancelled in the SPECT/CT cohort (9.3% vs. 2.5%; *p* < 0.0001). Head & neck, lower limb, and AJCC IB primaries were significantly less likely to proceed to SNB. SPECT/CT identified significantly more positive SNBs (20.9% vs. 16.5%; *p* = 0.038). SPECT/CT imaging was associated with improved disease-free (hazard ratio [HR] = 0.74; 95% confidence interval [CI]: 0.54–1.0); *p* = 0.048) and disease-specific survival (HR = 0.48; 95% CI: 0.3–0.78; *p* = 0.003). Patients who did not proceed to SNB had a significantly increased nodal relapse rate (23.5% vs. 6.8%; HR = 3.4; 95% CI: 1.9–6.2; *p* < 0.0001) compared with those who underwent SNB.

**Conclusions:**

This large cohort study confirms the increased accuracy of SPECT/CT for identifying SLN metastases, which would appear to have a significant therapeutic benefit, although an increased risk of cancellation of the SNB procedure on the day of surgery.

Primary cutaneous melanoma readily metastasises via the draining lymphatic system to the regional lymph nodes. Unlike other cancers, the lymphatic drainage pattern of the primary tumour cannot be easily predicted based on its anatomical location, making elective lymph node dissection an unreliable and ineffective treatment strategy. Sentinel node biopsy (SNB) is a technique developed to identify the lymphatic drainage of the primary tumour and to stage the regional lymph nodes to identify patients in need of further treatment.^1^ Since its development, SNB has evolved to become the standard of care for primary cutaneous melanoma and is incorporated in the current AJCC classification system.^2^

Accurate preoperative lymphoscintigraphy (LSG) is essential for successfully performing SNB.^3^ However, there is a recognised false-negative rate associated with the technique,^4^ particularly in the head and neck region, and data from Sydney suggest that failure to visualise the SN accurately is contributory to the false-negative rate.^5^ The introduction of coregistered single-photon emission computed tomography with integrated computed tomography (SPECT/CT) has greatly improved the accuracy of localisation of the sentinel lymph node(s) (SLN) prior for primary cutaneous melanoma.^6–11^ At a national SNB consensus meeting, concerns were raised by the pathologists that more SLNs were being harvested since the introduction for SPECT/CT imaging, thereby impacting on workload and throughput.^12^ Similarly, concerns were raised by the surgeons that the increasing accuracy of SLN identification, whilst generally considered a positive benefit, was associated also with increasing surgical morbidity and an increasing number of SNB procedures cancelled preoperatively. The purpose of this study was to investigate the effectiveness of SPECT/CT imaging for SNB staging of cutaneous melanoma in a large cohort treated at an academic, tertiary referral cancer centre.

## Methods

This is a single-centre, retrospective, cohort study based on a prospectively collected institutional database. Central regulatory approval for this study was granted by the UK NHS Health Research Authority (IRAS ID: 234471). Data were collected over a 10-year period, from 2009 to 2019, inclusive. The primary inclusion criteria were all adult patients (aged 18 years or older) with primary cutaneous melanoma AJCC T-stage pT1b-pT4b scheduled for wide excision and SNB at our regional cancer centre. All primary melanoma specimens and their associated wide excision and SNB specimens were subject to centralised pathology review as part of their standard of care. Patients with mucosal and/or genital melanomas, in addition to melanocytic tumours of unknown malignant potential (“mel-TUMP”) were excluded. Standard patient demographic data and primary tumour characteristics were recorded.

The primary outcome measures were the rates of successful preoperative sentinel node localisation and successful completion of the planned sentinel node biopsy compared between the two planning protocols. Subgroup analyses included demographics, tumour AJCC stage, and location. The number of sentinel nodes and their nodal basins identified by the imaging protocols were recorded. Second echelon nodes, identified by the reporting nuclear medicine clinician by comparing the early-phase and delayed imaging, were not included in these measurements. The reasons for cancellation also were assessed to see if there was a pattern that could be attributed to either imaging modality. The number of nodes harvested at the SNB procedure were *not* recorded, because this is a recognised weak correlation with the accuracy of the imaging protocol and was not the outcome of interest in this study.^13,14^ Survival outcomes data were collected from patient follow-up which included disease-free survival (DFS), nodal relapse-free survival (NRFS), disease-specific survival (DSS), and overall (OS) survival according to standard FDA criteria.^15^ DFS was subclassified as local (including local, satellite, and in transit recurrences), regional (within draining nodal field), or distant recurrences and were censored at the date of first diagnosis on the histopathology and/or radiology report. In the case of multiple site recurrence, DFS was recorded based on the first instance and highest stage at that time, according to the “first/worst” principle.

### Procedure/Technique

All SLNB procedures were performed at a single tertiary referral cancer centre according to a standardised international protocol using a dual localisation technique.^3^ Patients underwent a preoperative sentinel localisation and mapping using 20-40 MBq technetium-labelled nanocolloid (NanoColl™) injected intradermally directly adjacent to the centre of the melanoma scar. In all cases, patients were scanned sequentially using planar lymphoscintigraphy each minute for 10 minutes and then a separate delayed planar LSG scan was performed at 1 hour. From October 19, 2015, the patient underwent a second SPECT/CT (Siemens, Germany) scan, which coregistered the gamma signal to a whole body CT. For all patients, LSG images were available to the reporting nuclear medicine physician and the operating surgeon. After October 2015, both clinicians also had the additional information from the co-registered SPECT/CT. For the purpose of this study, patients were divided into two cohorts: those who had had only planar LSG imaging (pre October 19, 2015) and those who had both LSG and SPECT/CT. Surgery was performed in a standardised manner according to our unit protocol, using a dual-localisation technique of intraoperative injection of Patent Blue dye (Geurbet, France) and radiolocalisation using a Navigator 2.0 gamma probe (Dilon Technologies, Virginia, USA). Further technical details of our protocol have been described elsewhere.^16^

### Statistical Analysis

Pseudoanonymised data were analysed using Jamovi software (Version 1.6, Sydney, Australia https://www.jamovi.org) and R-Studio (version 1.3.1093, Boston, MA), both running R-language (version 3.6, https://cran.r-project.org/). Patients characteristics and histopathological parameters were summarised using descriptive statistics stratified by scan type. Differences between groups were tested using Kruskal-Wallis test as appropriate for continuous variables and Pearson chi-squared tests for categorical variables. Univariable and multivariable Cox proportional hazard regressions were performed to identify factors that are associated to either survival outcomes. Survival outcomes data were also analysed using the Kaplan-Meier log-rank test.

## Results

A total of 1522 primary cutaneous melanoma patients were identified from our prospective institutional database. Table 1 summarises the cohorts and highlights the outcome variables stratified by preoperative imaging modality (LSG versus SPECT/CT). The LSG group comprised 963 patients and the SPECT/CT group 559 patients. The table demonstrates that both groups were matched for age, site of primary, Breslow thickness, AJCC stage, incidence of ulceration, and microsatellites. There was significantly greater proportion of men in the SPECT/CT cohort (56.4% vs. 51.0%; *p* = 0.043)

**Table 1:** Patient demographics, tumour factors, imaging, and patient outcomes stratified by imaging modality

	N	LSG	SPECT/CT	Test statistic
		963	559	
Age (yr)	1522	51 (63–71)	53 (65–72)	F(1,1520) = 3.17, *p* = 0.075^c^
Gender: M	1522	51.0% (491/963)	56.4% (315/559)	χ^2^(1) = 4.09, *p* = 0.043
Primary site	1522			χ^2^(3) = 1.17, *p* = 0.761
*Torso*		37.9% (365/963)	39.0% (218/559)	
*Head and neck*		15.9% (153/963)	16.6% (93/559)	
*Upper extremity*		20.9% (201/963)	18.6% (104/559)	
*Lower extremity*		25.3% (244/963)	25.8% (144/559)	
Breslow thickness (mm)	1,522	1.10 (1.70–2.90)	1.20 (1.70-2.98)	F(1,1520) = 0.54, *p* = 0.461^c^
Ulceration: Yes	1,522	24.1% (232/963)	24.0% (134/559)	χ^2^(1) = 0.00, *p* = 0.958
Microsatellites: yes	1522	3.9% (38/963)	5.2% (29/559)	χ^2^(1) = 1.30, *p* = 0.255
AJCC stage^a^	1522			χ^2^(6) = 11.23, *p* = 0.081
*IB*		54.2% (522/963)	53.5% (299/559)	
*IIA*		20.8% (200/963)	19.1% (107/559)	
*IIB*		12.6% (121/963)	14.0% (78/559)	
*IIC*		8.5% (82/963)	8.2% (46/559)	
*IIIB*		2.0% (19/963)	2.5% (14/559)	
*IIIC*		2.0% (19/963)	2.7% (15/559)	
Basin count	1522	1 (1–2)	1 (1–2)	F(1,1520) = 3.63, *p* = 0.057^c^
Node count	1522	2 (1–3)	2 (3–4)	F(1,1520) = 66.54, *p* = 0.000^c^
SN status: positive	1446	16.5% (155/939)	20.9% (106/507)	χ^2^(1) = 4.31, *p* = 0.038
Site of first recurrence^b^	1522			χ^2^(3) = 47.94, *p* = 0.001
*None*		76.9% (741/963)	88.2% (493/559)	
*Local*		7.0% (67/963)	2.5% (14/559)	
*Regional*		3.7% (36/963)	5.4% (30/559)	
*Distant*		12.4% (119/963)	3.9% (22/559)	
SNB performed?: no	1522	2.5% (24/963)	9.3% (52/559)	χ^2^(1) = 34.58, *p* = 0.001

*N* number of nonmissing values; *SN* sentinel node; *SNB* sentinel node biopsy; *LSG* planar lymphoscintigraphy; *SPECT/CT* single positron emission computerised tomography with coregistered computerised tomography

^a^AJCC stage, 8th edition of the primary before SNB result

^b^Based on the first worst recurrence (see text)

^c^Kruskal-Wallis test

### Preoperative Sentinel Node Identification

Table 1 reveals that the median number of nodal basins identified was one (interquartile range [IQR] 1–2) for both imaging protocols. However, there was a significantly increased number of sentinel nodes identified by the SPECT/CT regimen compared with the LSG regimen (3 vs. 2; *p* < 0.0001). The analysis also showed a significantly increased sentinel node positivity rate in the SPECT/CT cohort (20.9% vs. 16.5%; *p* = 0.048). Multivariable analysis stratified for age, gender, Breslow thickness, ulceration and microsatellites identified the imaging modality as a significant independent predictor of sentinel node status (odds ratio 1.34 (range 1.0–1.78); *p* = 0.046).

### Effectiveness

SNB was performed in 95.0% (1446/1522) of all patients scheduled for the procedure. At least one sentinel node was identified and biopsied at operation in all but one patient (intraoperative failure rate = 0.6%). Table 2 shows that there was a significantly increased SNB cancellation rate in the SPECT/CT group compared with the LSG group (9.3% vs. 2.5%; *p* < 0.0001). The SPECT/CT group accounted for 68.4% (52/76) of all the SNB cancellations. Primary tumours located in the head and neck region or on lower extremity were more likely to be cancelled (38.2% and 35.5%, respectively) compared with those located on the upper limb or torso (9.2% and 17.1%, respectively; *p* < 0.0001). A significantly greater proportion of AJCC IB patients (T-Stage pT1b-pT2a) were cancelled in the SPECT/CT cohort (5.9% vs. 0.7%; Chi-square test for trend: *p* = 0.043). When the cancellation cohort were stratified by imaging modality, SPECT/CT imaging was associated with younger age (64 vs. 72 years, *p* = 0.023), thinner tumours (median Breslow thickness 1.3 mm vs. 2.8 mm, *p* = 0.16), and higher basin (2 vs. 1, *p* = 0.004) and node (6 vs. 1, *p* < 0.001) counts. The documented reasons for SNB cancellation were more likely to be due to too many nodes identified on the scan or inaccessible nodal location in the SPECT/CT group, whereas tracer migration failure was the most common reason for the LSG group (Chi-square test for trend; *p* = 0.003). Other reasons for canceling the SNB procedure included disease progression detected at time of imaging (including satellites at the primary site or unambiguous distant metastases seen on the SPECT/CT scan: *n* = 5), cancellation of procedure by patient request (*n* = 3), patient failure to attend (n = 2), and severe allergic reaction (*n* = 1). Cancellation was not associated with gender of the patient, nor the presence of microsatellites.

**Table 2:** Subgroup analysis of the cohort where SNB was not performed, stratified by imaging modality

	N	LSG	SPECT_CT	Test statistic
		24	52	
SNB performed: no	1522	2.5% (24/963)	9.3% (52/559)	χ^2^(1) = 34.58, *p* = 0.000
Reason for cancellation	76			χ^2^(3) = 13.76, *p* = 0.003
*Too many nodes*		29.2% (7/24)	65.4% (34/52)	
*No tracer migration*		50% (12/24)	17.3 (9/52)	
*Nodal location*		0% (0/24)	7.7% (4/52)	
*Other*		20.8% (5/24)	9.6% (5/52)	
Gender: M	76	45.8% (11/24)	50% (26/52)	χ^2^(1) = 0.114, *p* = 0.736
AGE	76	72 (63–77)	64 (56–71)	F(1,74) = 5.15, *p* = 0.023
Primary site				χ^2^(3) = 0.929, *p* = 0.819
*Torso*		16.7% (4/24)	17.3% (9/52)	
*Head and neck*		41.7% (10/24)	36.5% (19/52)	
*Upper extremity*		12.5% (3/24)	7.7% (4/52)	
*Lower extremity*		29.2% (7/24)	38.8% (20/52)	
Breslow thickness (mm)	76	2.8 (1.6–5.1)	1.3 (0.9-3.3)	F(1,74) = 5.82, *p* = 0.016
Ulceration: yes	76	41.7% (10/24)	21.2% (11/52)	χ^2^(1) = 3.46, *p* = 0.063
Microsatellites: yes	76	4.2% (1/24)	7.7% (4/52)	χ^2^(1) = 0.332, *p* = 0.564
AJCC stage^a^	76, 1522	2.5% (24/963)	9.3% (52/559)	χ^2^(5) = 34.58, *p* = 0.043
*IB*		29.2% (7/24)	63.5% (33/52)	
		0.7% (7/963)	5.9% (33/559)	
*IIA*		16.7% (4/24)	5.8% (3/52)	
		0.4% (4/963)	0.5% (3/559)	
*IIB*		16.7% (4/24)	9.6% (5/52)	
		0.4% (4/963)	0.9% (5/559)	
*IIC*		33.3% (8/24)	13.5% (7/52)	
		0.8% (8/963)	1.3% (7/559)	
*IIIB*		4.2% (1/24)	1.9% (1/52)	
		0.1% (1/963)	0.2% (1/559)	
*IIIC*		0% (0/24)	5.8% (3/52)	
		0% (0/963)	0.5% (3/559)	
Basin count	76	1 (0–1)	2 (1-2)	F(1,74) = 8.2, *p* = 0.004
Node count	76	1 (0–5)	6 (4-6)	F(1,74) = 11.4, *p* = 0.000

*N* number of nonmissing values

^1^Kruskal-Wallis test

^2^Pearson test

^a^AJCC stage, 8th edition of the primary before SNB result

### Survival Outcome

**Fig. 1:**
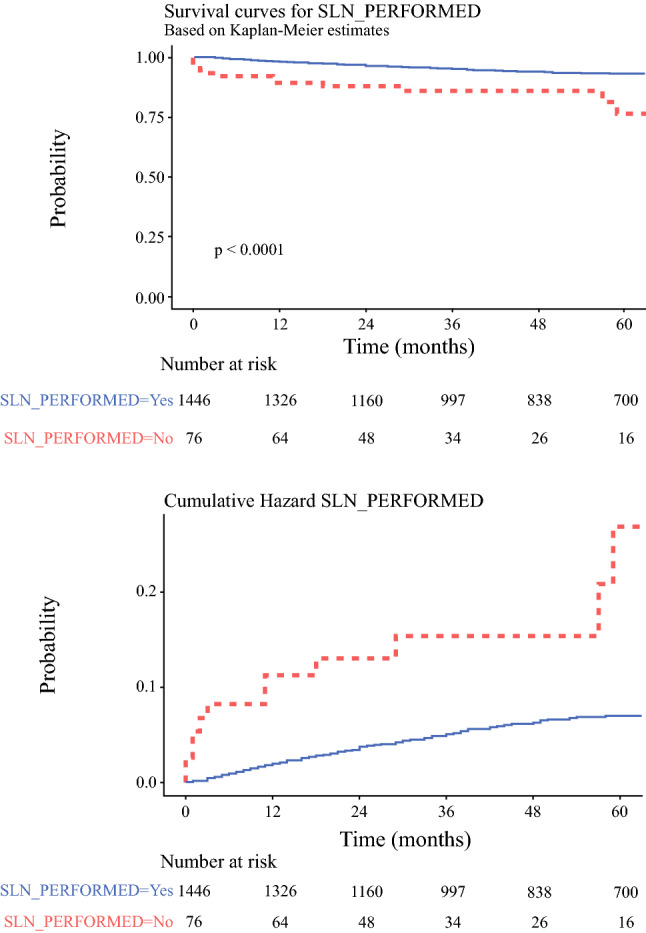
Kaplan-Meier cumulative hazard plots comparing nodal relapse-free survival in patients who underwent SNB compared with those whose procedures were cancelled

The median follow-up period was 85 months for the LSG group and 32 months for the SPECT/CT group. There were 288 recurrences and 155 melanoma deaths in total during the study period. Univariable analysis showed that patients who did not undergo sentinel node biopsy had a significantly worse 5-year nodal relapse-free survival (76.5% vs. 93.2%; HR = 3.4 (95% CI: 1.9–6.2); *p* < 0.0001; Fig. 1), but this did not translate into a significantly worse DSS during that period (HR = 0.5; *p* = 0.242). An intention to treat analysis of the cohort demonstrated a significantly increased risk of nodal relapse in the SPECT/CT group (HR = 1.55 (95% CI: 1.0–2.4); *p* = 0.049) and reduced risk of death from melanoma (HR = 0.60 (95% CI: 0.38–0.95); *p* = 0.03) compared with the LSG group but no significant differences in DFS.

**Fig. 2:**
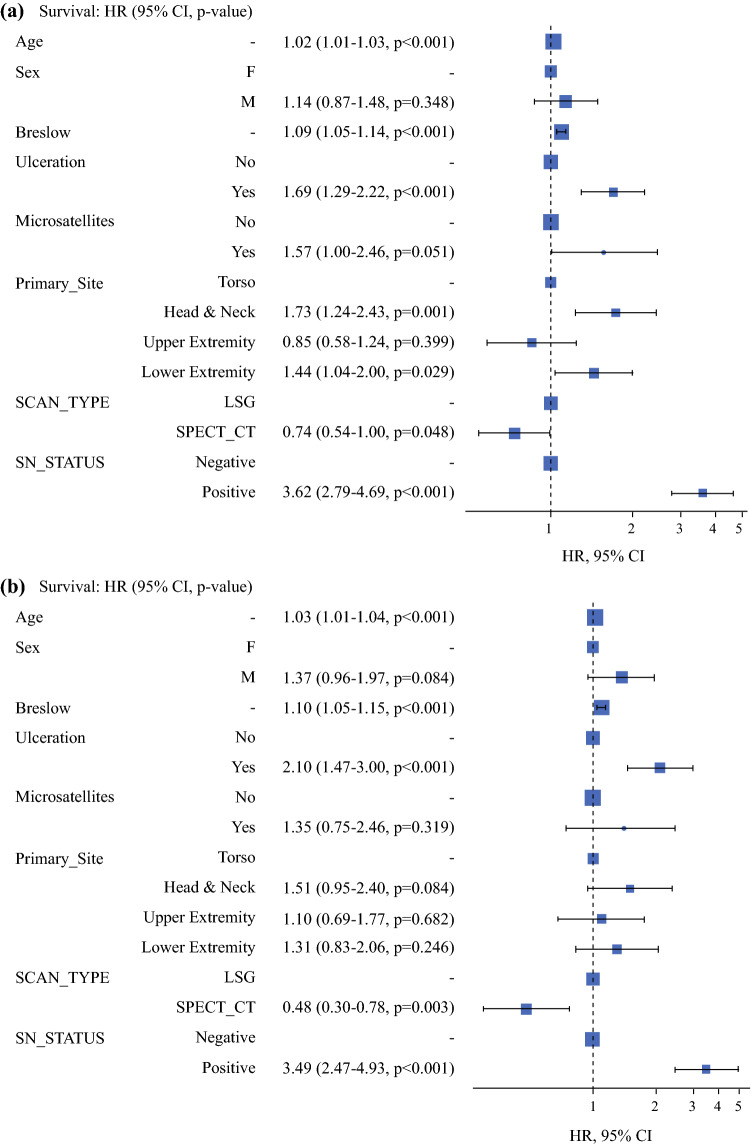
**a** Disease-free survival. **b** Disease-specific survival

Patients who did not proceed to SNB were excluded from the remaining analysis to test the effect of the imaging modalities on patient outcome. Univariable analysis showed no significant difference in DFS (HR = 0.82 (95% CI: 0.61–1.11); *p* = 0.21) but a significant difference in DSS (HR = 0.60 (95% CI: 0.37–0.96); *p* = 0.031) with an absolute survival difference of 3.4% at 3 years, in favour of the SPECT/CT group. The nodal relapse rates were the same for both cohorts, when SNB was performed (HR = 1.34 (95% CI: 0.83–2.16); *p* = 0.22). Multivariable analysis, stratifying for age, gender, Breslow thickness, ulceration and microsatellites, primary site location, and sentinel node status revealed that the imaging modality used was a significant independent predictor of DFS (HR = 0.74 (95% CI: 0.54–1.0); *p* = 0.048) and DSS (HR = 0.48 (95% CI: 0.3–0.78); *p* = 0.003) but not of nodal relapse-free survival (HR = 1.26 (95% CI: 0.78–2.04); *p* = 0.34).These findings are represented graphically in the odds ratio plots (Fig. 2a-c).

## Discussion

The prognostic significance of SN status for primary cutaneous melanoma is undoubted.^2,17^ Furthermore, the MSLT-1 data indicated that a nodal micrometastasis without intervention is likely to progress to palpable disease and that the act of removing the lymph nodes at the time of SNB may prevent further nodal progression and relapse.^17^ However, the main role of sentinel node biopsy has shifted from identifying patients who require surgical intervention, namely regional lymphadenectomy, to accurately identifying patients who are eligible for adjuvant systemic therapy.^12,18^ The dataset that underpinned the latest AJCC classification system for melanoma, confirmed the primacy of SNB for staging primary cutaneous melanoma.^2^ In addition, maturing data from large phase III clinical trials are suggesting a long-term survival benefit from adjuvant systemic therapy.^19,20^ Accordingly, the accurate identification of SLNs has taken on an additional significance in the past few years.

The EANM guidelines suggest that SPECT/CT is preferable on the basis that it improves the localization of SLNs and reduces misinterpretation of images, because they are three-dimensional and have better contrast and spatial resolution.^21^ It is suggested that this is particularly important in the head and neck region.^22^ The EANM guidelines also suggest that SPECT/CT is highly recommended for the groin and axillary areas, because “…it facilitates the detection of in-transit nodes and aberrant lymphatic drainage stasis in lymph vessels and consequently facilitates the surgical procedure.” A large, prospective, multicentre trial, the International Atomic Energy Agency Sentinel Node Trial, demonstrated that SPECT/CT had modified the surgical approach in 37% of patients with melanoma, with greatest improvement in accuracy seen in the head and neck, and truncal regions.^23^ A recent meta-analysis demonstrated a higher overall SLN detection rate and proportion of patients with additional SLNs.^24^ Another study highlighted the health economic benefit of this approach.^25^ Our data are consistent with the previous literature in that we found a significantly increased number of SLNs and were identified by SPECT/CT compared with LSG (median 3 vs. 2; *p* < 0.0001) (Table 1). Furthermore, a near-significant trend of an increase in the number of identified nodal basins also was found (*p* < 0.06) (Table 1).

### Perioperative Outcomes and Effectiveness

Whilst our data have confirmed previous findings that the additional information provided by SPECT/CT imaging of SLNs increases the opportunity for perioperative decision-making, but perhaps not always in the positive manner that previous publications have suggested.^21^ Our data have shown that the tracer agent migration failure rate has been consistent across the study period (1–1.5%), regardless of the imaging technique employed. However, there was a significantly increased number of preoperative cancellations of the SNB procedure in the SPECT/CT group compared with the LSG group (9.3% vs. 2.5%; *p* = 0.003). Closer inspection of Table 2 highlights that the two main reasons were too many nodes were identified or the relative surgical inaccessibility of the SLNs located on the scan. It is clear that these decisions are subjectively made, based on both the patients’ and surgeons’ evaluations of the perceived risk of missing the diagnostic opportunity from not undertaking the SNB versus the benefit from avoiding the potential morbidity of the procedure and, secondarily, the opportunity to avoid a general anaesthetic if a wide excision alone is performed instead. Whilst these decisions were subjective, no gender bias was detected in our analysis.

Further inspection of the data provides clues to the main factors that influence that decision-making process. In the SPECT/CT group, cancellations of the SNB procedure were significantly more common in the AJCC IB group (pT1b-pT2a), where the risk of sentinel node positivity is relatively low (~5–10%). In addition, cancellations were significantly more common in the head and neck and lower extremity regions. Whilst the granular details of the decision-making process are unavailable in this study, it is reasonable to assume that the reasons for cancellation are due to the anatomical idiosyncrasies of both these regions. In the head and neck, the lymphatic drainage is usually complex, and often bilateral.^26^ The combined risks of potential injury to superficial cranial nerves, drainage to multiple levels in the neck occasionally necessitating several incisions for access, and the reduced accuracy of SLN localisation in general make SNB relatively undesirable and hazardous in this region.^4,26^ Similarly, lower-extremity melanomas routinely drain to the pelvis, which can be challenging for the surgeon to access to perform a successful procedure.^26^ Furthermore, dual drainage to the groin and pelvis significantly increases the risk of postoperative lymphoedema, which is a major quality of life and survivorship issue for melanoma patients, where the majority of patients are sentinel node-negative and nearly half of the patients are younger than aged 60 years.^27,28^

### Survival Outcomes

The MSLT-1 study is rightly described as a landmark trial, which confirmed the prognostic and therapeutic utility of SNB for primary cutaneous melanoma.^17,29^ Our data are aligned with several of the main outcomes of the study, including a reduced disease-specific survival for the SNB-positive patients and a reduced regional control rate in the patients who did not undergo sentinel node biopsy. Our data showed an increased SNB-positive rate in the SPECT/CT cohort (20.9% vs. 16.5%; *p* = 0.048). The MSLT-1 study data made a highly compelling argument for a survival benefit for a small group of patients undergoing SNB who have their focus of micrometastatic disease excised. Our data showed that, despite the increased incidence of sentinel node positivity, the SPECT/CT cohort had a significantly improved disease-specific survival compared with the LSG cohort. Whilst these data need to be interpreted with caution, one possible explanation for the observed outcome is the more accurate identification of sentinel nodes containing the metastatic focus and their subsequent removal afforded by the SPECT/CT imaging regimen.

A potentially counterintuitive finding was the significantly increased risk of nodal relapse in the SPECT/CT group from the intention to treat analysis (HR = 1.55 (95% CI: 1.0–2.4); *p* = 0.049), despite the improved accuracy of the technique and the increased SNB positivity rate. The likely explanation is the effect of the significantly increased risk of perioperative cancellation of the SNB in this cohort compared with the LSG cohort, given the targeted subgroup analysis showed no difference in this endpoint when the cancelled patients were excluded. We believe this is a hitherto unreported negative consequence of the preferential use of SPECT/CT imaging and is an important point to consider when patients are being counselled for their surgery, particularly when considering cancelling the SNB procedure. It also is important to note, however, that this did not translate into worse DSS, in contrast to the results of the MSLT-1 study.^17^

### Study Limitations

We acknowledge several limitations of our study. Our data are limited to a single centre, albeit that it is one of the largest cohorts to report on this subject. Furthermore, the follow-up is relatively short and is therefore unable to detect the effects of late recurrences beyond 5 years, which are common in cutaneous melanoma.^30^ The major limitation of the study is that the cohorts are not contemporaneous. During the period 2009–2015, when planar LSG was the only imaging modality available, patients were routinely offered completion lymph node dissection (CLND) for a positive SNB, although our centre was actively recruiting to the MSLT-2 study,^31^ and it was therefore not universally applied. Subsequently, the results of that study confirmed that CLND was not effective for SNB positive patients and no longer offered as a standard of care for our patients. From late 2016 onwards, effective systemic therapy became available for patients with recurrences or high-risk disease. Accordingly, it is challenging to draw major conclusions and the comparative DFS and DSS outcomes between the two cohorts should be interpreted with caution, although the two cohorts were otherwise well-matched in terms of patient demographics and tumour characteristics otherwise, which potentially limits the effects of these biases.

## Conclusions

This large cohort study confirms the increased accuracy of SPECT/CT for identifying SLN metastases in cutaneous melanoma, which is associated with a significant therapeutic benefit in terms of improved disease-free and disease-specific survival. However, the improved accuracy comes with an increased workload for pathology departments and an increased risk of cancellation of the SNB procedure on the day of surgery, which in turn has a negative impact on nodal relapse-free survival. These data would suggest evaluating the true effectiveness of SPECT/CT imaging in SNB staging of melanoma is complex and merits further investigation.

## References

[1] Morton DL, Wen DR, Wong JH (1992). Technical details of intraoperative lymphatic mapping for early stage melanoma. Arch Surg..

[2] Gershenwald JE, Scolyer RA, Hess KR, et al. Melanoma staging: Evidence-based changes in the American Joint Committee on Cancer eighth edition cancer staging manual: Melanoma Staging: AJCC 8th Edition. *CA Cancer J Clin*. 2017;67:472–92. 10.3322/caac.21409 PMC5978683 29028110

[3] Wong SL, Faries MB, Kennedy EB (2018). Sentinel lymph node biopsy and management of regional lymph nodes in melanoma: American society of clinical oncology and society of surgical oncology clinical practice guideline update. J Clin Oncol..

[4] Morton DL, Cochran AJ, Thompson JF, et al. Sentinel node biopsy for early-stage melanoma: accuracy and morbidity in MSLT-I, an international multicenter trial. *Ann Surg*. 2005;242:302–11; discussion 311–3. 10.1097/01.sla.0000181092.50141.fa PMC1357739 16135917

[5] Karim RZ, Scolyer RA, Li W (2008). False negative sentinel lymph node biopsies in melanoma may result from deficiencies in nuclear medicine, surgery, or pathology. Ann Surg..

[6] Uren RF (2009). SPECT/CT Lymphoscintigraphy to locate the sentinel lymph node in patients with melanoma. Ann Surg Oncol..

[7] Klode J, Poeppel T, Boy C (2011). Advantages of preoperative hybrid SPECT/CT in detection of sentinel lymph nodes in cutaneous head and neck malignancies. J Eur Acad Dermatol Venereol..

[8] Doepker MP, Yamamoto M, Applebaum MA (2017). Comparison of Single-Photon Emission Computed Tomography-Computed Tomography (SPECT/CT) and Conventional Planar Lymphoscintigraphy for Sentinel Node Localization in Patients with Cutaneous Malignancies. Ann Surg Oncol..

[9] Trinh BB, Chapman BC, Gleisner A (2018). SPECT/CT adds distinct lymph node basins and influences radiologic findings and surgical approach for sentinel lymph node biopsy in head and neck melanoma. Ann Surg Oncol..

[10] Veenstra HJ, Vermeeren L, Olmos RAV (2012). The additional value of lymphatic mapping with routine SPECT/CT in unselected patients with clinically localized melanoma. Ann Surg Oncol..

[11] van der Ploeg IMC, Valdés Olmos RA, Nieweg OE (2007). The additional value of SPECT/CT in lymphatic mapping in breast cancer and melanoma. J Nucl Med..

[12] Peach H, Board R, Cook M (2020). Current role of sentinel lymph node biopsy in the management of cutaneous melanoma: a UK consensus statement. J Plast Reconstr Aesthet Surg..

[13] McMasters KM, Noyes RD, Reintgen DS (2004). Lessons learned from the Sunbelt Melanoma Trial. J Surg Oncol..

[14] Hudak KA, Hudak KE, Dzwierzynski WW (2015). Sentinel lymph node biopsy for melanoma: is there a correlation of preoperative lymphatic mapping with sentinel lymph nodes harvested?. Ann Plast Surg..

[15] Brody T. Clinical Trials: Study Design, Endpoints and Biomarkers, Drug Safety, and FDA and ICH Guidelines. Elsevier Science; 2016.

[16] Moncrieff MD, O’Leary FM, Beadsmoore CJ (2020). Effect of delay between nuclear medicine scanning and sentinel node biopsy on outcome in patients with cutaneous melanoma. Br J Surg..

[17] Morton DL, Thompson JF, Cochran AJ (2014). Final trial report of sentinel-node biopsy versus nodal observation in melanoma. N Engl J Med..

[18] Melanoma: Cutaneous. *NCCN Clinical Practice Guidelines in Oncology*. Available at: https://www.nccn.org/professionals/physician_gls/pdf/cutaneous_melanoma.pdf. 2021. Accessed 10 Apr 2021.

[19] Weber J, Mandala M, Del Vecchio M (2017). Adjuvant Nivolumab versus Ipilimumab in Resected Stage III or IV Melanoma. N Engl J Med..

[20] Dummer R, Hauschild A, Santinami M (2020). Five-year analysis of adjuvant Dabrafenib plus Trametinib in Stage III melanoma. N Engl J Med..

[21] Bluemel C, Herrmann K, Giammarile F (2015). EANM practice guidelines for lymphoscintigraphy and sentinel lymph node biopsy in melanoma. Eur J Nucl Med Mol Imaging..

[22] Nielsen KR, Chakera AH, Hesse B (2011). The diagnostic value of adding dynamic scintigraphy to standard delayed planar imaging for sentinel node identification in melanoma patients. Eur J Nucl Med Mol Imaging..

[23] Jimenez-Heffernan A, Ellmann A, Sado H (2015). Results of a prospective multicenter international atomic energy agency sentinel node trial on the value of SPECT/CT over planar imaging in various malignancies. J Nucl Med..

[24] Quartuccio N, Garau LM, Arnone A, et al. Comparison of 99mTc-Labeled Colloid SPECT/CT and Planar Lymphoscintigraphy in Sentinel Lymph Node Detection in Patients with Melanoma: A Meta-Analysis. *J Clin Med Res*.;9 . Epub ahead of print June 2, 2020. DOI: 10.3390/jcm9061680. 10.3390/jcm9061680 PMC7356992 32498217

[25] Stoffels I, Müller M, Geisel MH (2014). Cost-effectiveness of preoperative SPECT/CT combined with lymphoscintigraphy vs. lymphoscintigraphy for sentinel lymph node excision in patients with cutaneous malignant melanoma. Eur J Nucl Med Mol Imaging..

[26] Reynolds HM, Walker CG, Dunbar PR (2010). Functional anatomy of the lymphatics draining the skin: a detailed statistical analysis. J Anat..

[27] Faries MB, Thompson JF, Cochran A (2010). The impact on morbidity and length of stay of early versus delayed complete lymphadenectomy in melanoma: results of the multicenter selective lymphadenectomy trial (I). Ann Surg Oncol..

[28] Melanoma skin cancer statistics. *Cancer Research UK*. Available from: https://www.cancerresearchuk.org/health-professional/cancer-statistics/statistics-by-cancer-type/melanoma-skin-cancer. 2019. Accessed 30 Dec 2019.

[29] Bello DM, Faries MB (2020). The landmark series: MSLT-1, MSLT-2 and DeCOG (Management of Lymph Nodes). Ann Surg Oncol..

[30] Faries MB, Steen S, Ye X, et al. Late recurrence in melanoma: clinical implications of lost dormancy. *J Am Coll Surg*. 2013;217:27–34; discussion 34–6. 10.1016/j.jamcollsurg.2013.03.007 PMC3731060 23643694

[31] Faries MB, Thompson JF, Cochran AJ (2017). Completion dissection or observation for sentinel-node metastasis in melanoma. N Engl J Med..

